# Screen-based sedentary behaviors but not total sedentary time are associated with anxiety among college students

**DOI:** 10.3389/fpubh.2022.994612

**Published:** 2022-10-20

**Authors:** Tao Huang, Kefeng Zheng, Shiyuan Li, Yanxiang Yang, Lingxuan Kong, Ying Zhao

**Affiliations:** ^1^Department of Physical Education, Shanghai Jiao Tong University, Shanghai, China; ^2^Chair of Sport and Health Management, Technical University of Munich, Munich, Germany

**Keywords:** sedentary behaviors, screen time, mental health, college students, anxiety

## Abstract

**Objective:**

The purpose of the study was to investigate the associations of device-measured total sedentary time and screen-based sedentary time with anxiety in college students.

**Methods:**

Three hundred and twenty-one college students (mean age = 19.72 ± 1.18, 55.8% females) were recruited from Shanghai, China. Total sedentary time was objectively measured using accelerometry, while screen-based sedentary time was self-reported. Anxiety symptom was evaluated using the Self-Rating Anxiety Scale. Linear regression modeling was used to assess the associations of total sedentary time and screen-based sedentary time with anxiety symptom.

**Results:**

Accelerometer-assessed total sedentary time was not associated with anxiety symptom. Prolonged sedentary time on TV and movie viewing (>2 h on weekdays) and social media using (>2 h on weekdays and weekend) were associated with a higher level of anxiety. However, time on video gaming and recreational reading was not associated with anxiety symptom.

**Conclusion:**

The findings indicated that screen-based sedentary behaviors but not total sedentary time were associated with anxiety symptom among college students. The associations of screen-based sedentary behaviors with anxiety symptom varied by the types of screen time.

## Introduction

Evidence showed that sedentary time among college students has increased over the past decade, and college students engage in higher level of sedentary time compared to the general young adult population (1, 2). Excessive time spent on sedentary behaviors has emerged as a potential yet modifiable risk factor for health and wellbeing (3–5). Sedentary behaviors refer to any waking behaviors with an energy expenditure of <1.5 metabolic equivalent units (METs) in either a sitting, reclining, or lying postures (6). World Health Organization guidelines on physical activity and sedentary behavior recommends that adults should limit the amount of time spent being sedentary (7). Mounting evidence demonstrated that prolonged time on sedentary behaviors is associated with an increased risk of a number of cardiometabolic diseases, premature death, and some types of cancer (8–11).

However, studies examining the associations of sedentary behaviors with mental health issues yielded inconsistent findings. Specifically, some epidemiological studies found that sedentary time was negatively associated with mental health symptoms, such as anxiety, stress, and depression (12, 13), whereas others observed no associations between certain measures of sedentary behaviors and mental health (14, 15). The inconsistent findings regarding sedentary time and mental health may partly attribute to the varied measurement of sedentary behaviors. Time spent on sedentary behaviors can be objectively assessed by wearable devices or subjectively self-reported (e.g., self-reported screen-based sedentary behaviors). Screen-based sedentary behavior refers to time spent using a screen-based device (e.g., smartphone, tablet, computer, television) while being sedentary in any context (e.g., school, work, recreational) (6). Given the fact that electronic screen devices become an unavoidable part of daily life activities, it is important to clarify the effects of different types of screen-based sedentary time on mental health. Indeed, recent studies on youth have suggested that the contents or types of screen-based sedentary time can be moderators for the association between sedentary time and mental health indicators (16–20). For example, Boers and colleagues found that time on social media using and television enhanced the symptoms of depression in adolescents (17). In a large-scale observational study, Kidokoro et al. observed that excessive sedentary time on newer types of screen behaviors (e.g., social media, online gaming, and online videos) was associated with higher risk of depression in children and adolescents, whereas time on television was associated with lower risk of depression (20). Regarding the mechanisms, there are three main theories existing in the literature explaining the varied effects of screen viewing on mental health (17). The upward social comparison hypothesis and reinforcing hypothesis suggest that the effects of screen time on mental health depend on the content of the screen media (20, 21). In contrast, the displacement hypothesis posits that all screen-based sedentary time has deleterious effects on mental health, since it replaces time on other activities such as physical activity and sleep (17).

College students face academic, social, and other challenges, and recent evidence suggested that the prevalence of mental health issues hence increased in college students (22, 23). Anxiety is a common mental health indicator in this population. Anxiety disorders have severely deleterious effects on social, occupational, and other areas of functioning (24). Although previous studies have provided some evidence on the negative associations of prolonged sedentary time on some mental health indicators (25, 26), it remains to elucidate the associations of different types of screen-based sedentary behaviors with anxiety in colleges students. Therefore, the current study measured both device-assessed and self-reported sedentary time in order to clarify the associations of total sedentary time and screen-based sedentary time with anxiety symptom in college students.

## Methods

### Participants

The cross-sectional study was conducted at a university in Shanghai, China. The college students were informed of the opportunity to participate in this study by their physical education class teachers and advertisements on campus. In total, 321 college students (55.8% females) volunteered to participate in the study and provided complete data. All of them provided written informed consent. Ethical approval was obtained from the Institutional Review Board for Human Research Protections at Shanghai Jiao Tong University.

### Measure

#### Screen-based sedentary behaviors

Screen-based sedentary behaviors were self-reported using a questionnaire. The participants were required to report content-specific screen-based recreational behaviors on weekdays and weekend, including daily time on movie/TV viewing, social media using, recreational reading, and video gaming. For movie/TV viewing, the participants were asked “How much time do you usually spend on movie/TV viewing (including TV, movie, short video, etc.) per day?” For social media using, the participants were asked “How much time do you usually spend on social media using (including WeChat, QQ, Weibo, etc.) per day?” For social recreational reading, the participants were asked “How much time do you usually spend on recreational reading (including reading online novels, browsing websites, etc.) per day?” For video gaming, the participants were asked “How much time do you usually spend on video gaming per day?” The responses on each screen-based sedentary time were divided into three categories (i.e., no more than 1 h, 1–2 h, and more than 2 h).

#### Device-assessed sedentary time

Total sedentary time was objectively measured using the Axivity AX3 (Axivity Ltd., Newcastle, UK) wrist-worn triaxial Accelerometer. The AX3 was worn 24 h for 7 consecutive days, where the 24-h protocol has been shown with higher wear-time compliance compared to the waking-hour protocol (27, 28). The participants were instructed not to remove the AX3 at any time during the measurement periods. If removed, they were instructed to note the incident. The AX3 was set up to record tri-axial acceleration data at a sampling frequency of 100 Hz (default) with a dynamic range of ±8 g (default).

The R-based GGIR package (https://cran.r-project.org/web/packages/GGIR/, version 2.4-0) was used to process the raw AX3 data (29). Specifically, the metric ENMO (Euclidean Norm Minus One with negative values rounded to zero) was used to calculate the raw acceleration data, with the average levels per five-second-epochs (30). Furthermore, we defined the accepted participants in the analysis as those with a minimum record of 16 h/day for 4 days (3 weekdays and 1 weekend) (31). The non-wear time during valid days was defined based on a previously established algorithm (32). The cut points of sedentary behavior for adults (< 1.5 MET: 0–99 CPM; an average ENMO below 45 mg) were applied (30, 32), maintaining the vertical axis value over the counts. The data processing R codes are available from the authors on request.

#### Anxiety

Anxiety symptom was evaluated using the Self-Rating Anxiety Scale (SAS) (33). The SAS is a widely-used, 20-item screening tool for anxiety symptoms. Its Chinese version has been evaluated in Chinese population and showed good reliability and validity (34). The participants were requested to respond on a 4-point Likert-type scale (1 = none or a little of the time, 2 = some of the time, 3 = a good part of the time, 4 = most or all of the time). The total crude score ranges from 20 to 80. A higher score indicates greater tendency to anxiety. The Cronbach's alpha coefficient for the current study is 0.98.

#### Statistical analyses

Potential sex differences in participants' characteristics were evaluated by independent sample T-Test. The associations between different measures of sedentary time and mental health indicators were analyzed using multiple linear regression modeling. Time on screen-based sedentary behaviors were transformed into three-level categorical variables (i.e., ≤1 h, 1–2 h, and > 2 h). Model assumptions of normality of residuals and multicollinearity were checked by quantile-quantile plots and variance inflation factor, respectively. No violations of model assumption were detected. The standardized β coefficient was reported. The statistical analysis was performed using a commercial statistical package SPSS (IBM, Armonk, NY). The null hypothesis was rejected for two-sided values of *p* < 0.05. The graphical illustration of results was made using GraphPad Prism (GraphPad Software, San Diego, CA).

## Results

Participants' characteristics stratified by sex are presented in Table 1. Females have higher score of anxiety than males (*p* < 0.05). No differences were observed in total sedentary time between males and females (*p* > 0.05). There were significant differences in objectively measured physical activity by sex (*p* < 0.05). Male students spent more time on moderate-to-vigorous physical activity (MVPA) than female students (*p* < 0.05).

**Table 1:** Characteristics of participants.

	**Males**	**Females**	**All**	***p* for sex**
	**(*n* = 142)**	**(*n* =179)**	**(*n* =321)**	
Age (years)	19.79 ± 1.25	19.66 ± 1.11	19.72 ± 1.18	0.33
Anxiety	31.29 ± 7.31	32.97 ± 7.40	32.22 ± 7.40	**<0.05**
Total sedentary time (min/day)	739.76 ± 38.36	727.14 ± 71.15	732.72 ± 70.10	0.11
MVPA (min/day)	69.84 ± 31.21	66.96 ± 23.23	68.23 ± 27.05	**<0.05**

MVPA, moderate- to- vigorous physical activity. Data are expressed as mean and standard deviation. Bold values represent statistically significant *p*-values (*p* < 0.05).

As shown in Table 2, the accelerometer-assessed total sedentary time was not significantly associated with the score of anxiety in both models (both *p* > 0.05). The total sedentary time on weekdays was not significantly associated with the score of anxiety in both models (both *p* > 0.05). Similarly, the total sedentary time on weekends was not significantly associated with the score of anxiety in both models (both *p* > 0.05). There were no significant differences on the associations of total sedentary time with anxiety between females and males.

**Table 2:** Associations between objectively-measured total sedentary time and anxiety.

	**Anxiety**	
	**Model 1 β (95 % CI)**	**Model 2 β (95 % CI)**
Total sedentary time of weekdays (min/day)	−0.009 (−0.019–0.001)	−0.009 (−0.019–0.0004)
Total sedentary time of weekend (min/day)	−0.004 (−0.012–0.003)	−0.005 (−0.012–0.003)
Total sedentary time (min/day)	0.001 (−0.012–0.011)	−0.002 (−0.015–0.01)

Analysis was conducted by separate multiple linear regressions. Model 1: adjusted for sex, age and mother's education; Model 2: adjusted for model 1 + objectively measured moderate- to- vigorous physical activity.

**Figure 1:**
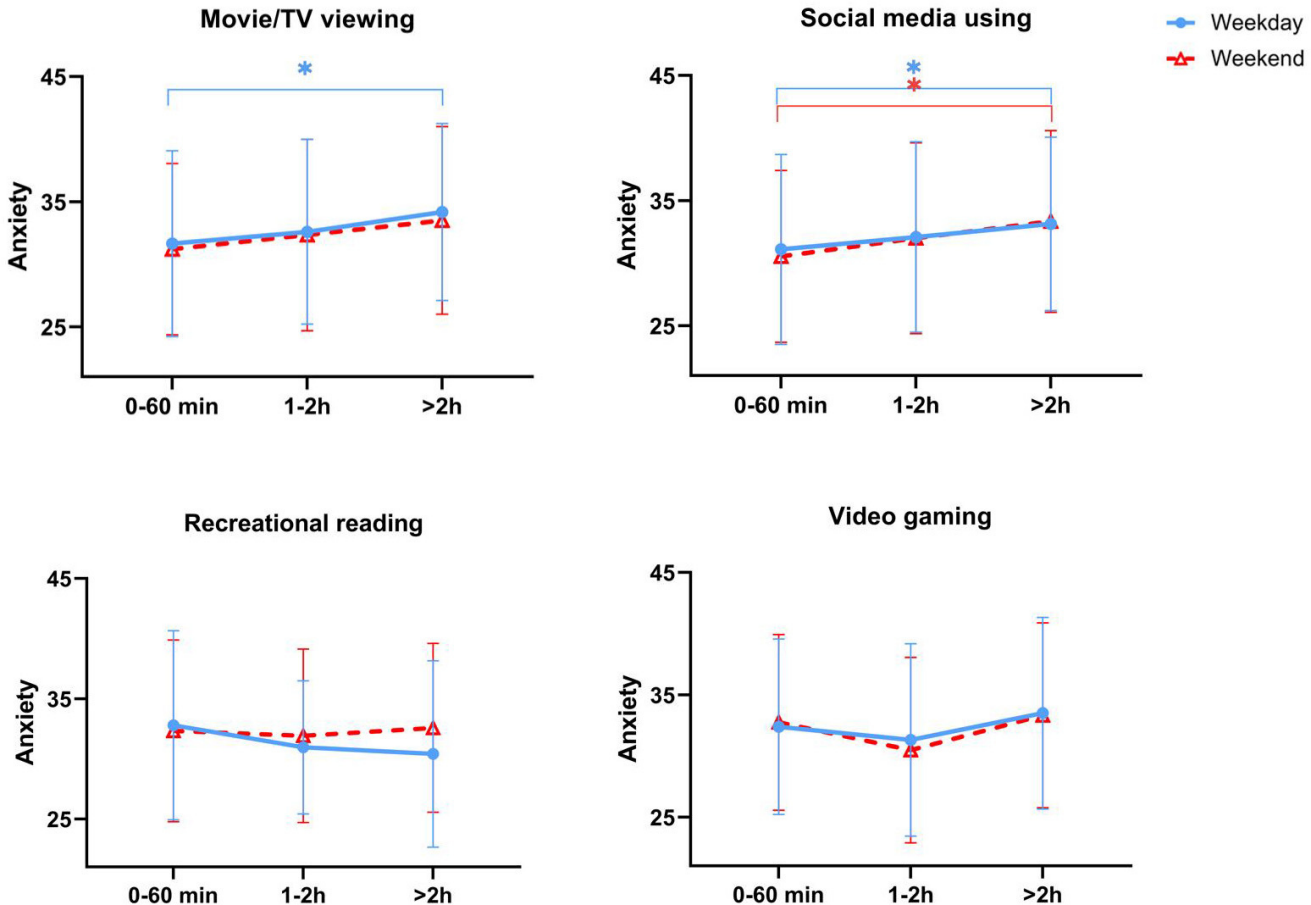
Trends of anxiety scores with time spent on different screen-based sedentary behaviors. Data are expressed as mean and standard deviation. * indicates *p* < 0.05 compared to reference.

With regard to the time spent on the four screen-based sedentary behaviors, there were differences on movie/TV viewing and video gaming between weekdays and weekend (both *p* < 0.05). However, there were no significant difference on social media using and recreational reading between weekdays and weekend. The results of the associations between screen-based sedentary behaviors and anxiety symptom are shown in Table 3. Time spent on video gaming and recreational reading (both on weekdays and weekend) were not associated with anxiety (both *p* > 0.05). Compared to movie/TV viewing <1 h/day, spending more than 2 h on movie/TV viewing on weekdays was positively associated with anxiety after adjusting for sex, age and mother's education (*p* < 0.05), and the association persisted with further adjustment for objectively-measured MVPA (*p* < 0.05). Meanwhile, on both weekdays and weekend, compared to social media using <1 h/day, spending more than 2 hours on social media was positively associated with anxiety after adjusting for sex, age and mother's education (*p* < 0.05), and the association persisted with further adjustment for objectively-measured MVPA (*p* < 0.05). The results indicated that longer time (>2 h/day) spent on movie/TV viewing and social media was correlated with higher anxiety scores (also see Figure 1).

**Table 3:** Associations of screen-based sedentary behaviors during weekdays and weekend with anxiety.

	**Anxiety (weekdays)**		**Anxiety (weekend)**	
	**Model 1 β (95 % CI)**	**Model 2 β (95 % CI)**	**Model 1 β (95 % CI)**	**Model 2 β (95 % CI)**
Movie/TV viewing (h/day)				
≤1 h (Ref.)	-	-	-	-
1 – 2 h	1.46 (−0.36–3.28)	1.44 (−0.38–3.27)	1.22 (−0.64–3.09)	1.19 (−0.68–3.05)
> 2h	**1.39 (0.02–2.76)**	**1.38 (0.004–2.75)**	0.85 (−0.31–2.00)	0.88 (−0.28–2.03)
Social media (h/day)				
≤1 h (Ref.)	-	-	-	-
1 – 2 h	1.54 (−0.58–3.65)	1.58 (−0.54–3.70)	1.46 (−0.75–3.68)	1.45 (−0.77–3.67)
> 2h	**1.25 (0.15–2.35)**	**1.25 (0.15–2.36)**	**1.39 (0.27–2.51)**	**1.40 (0.28–2.52)**
Video gaming (h/day)				
≤1 h (Ref.)	-	-	-	-
1 – 2 h	0.07 (−2.10–2.25)	0.09 (−2.09–2.27)	−1.11 (−3.23–1.02)	−1.18 (−3.31–0.95)
> 2h	1.23 (−0.40–2.85)	1.21 (−0.41–2.84)	0.90 (−0.43–2.22)	0.88 (−0.44–2.21)
Recreational reading (h/day)				
≤1 h (Ref.)	-	-	-	-
1– 2 h	−1.64 (−3.53–0.25)	−1.70 (−3.60–0.20)	−0.31 (−2.13–1.52)	−0.35 (−2.17–1.48)
> 2h	0.82 (−0.04–1.67)	0.81 (−0.04–1.67)	0.35 (−2.17–1.48)	0.24 (−1.33–1.80)

Analyses were conducted by separate multiple linear regressions. Values are standardized β and 95% CI. *p*-values less than 0.05 are set in bold. Model 1: adjusted for sex, age and mother's education; Model 2: adjusted for model 1 + objectively measured moderate- to- vigorous physical activity. Bold values represent statistically significant coefficients. Ref.: Reference.

## Discussion

This study examined the associations of accelerometer-assessed total sedentary time and screen-based sedentary behaviors with anxiety symptom in college students. The findings indicated that accelerometer-assessed total sedentary time was not associated with anxiety symptom. Screen-based sedentary time was associated with anxiety symptom. However, the associations of screen-based sedentary behaviors with anxiety symptom varied by the type of screen time. Longer time on TV and movie viewing and social media using was associated with higher level of anxiety.

Currently, only a few existing studies examined the associations of objectively measured total sedentary time with anxiety. In this study, the accelerometer-assessed total sedentary time both on weekdays and weekends was not associated with anxiety in college students. The current findings are consistent with a recent study which showed that accelerometer-assessed sedentary time was not associated with stress and anxiety in college students, however, self-reported sitting time on weekend was associated with higher trait anxiety and perceived stress levels (14). Similarly, another study also showed that device-assessed total sitting time and prolonged sitting time were not associated with anxiety in adults (15). In contrast, a study in college students showed that self-reported total sitting time was associated with higher levels of stress, anxiety, and depression (12). A previous review indicated that sedentary behaviors had a small yet positive association with anxiety, however, this review included studies measuring both screen-based sedentary behaviors and total sedentary time (26).

Most of previous studies examining screen-based sedentary behaviors did not clarify the effects of different types or contents of screen viewing on health indicators. Recent studies in children and adolescents shed light on the type-specific associations of screen time with mental wellbeing and cognitive function (17, 18, 20, 35). Although the findings are still mixed, those studies indicated that screen type moderated the strength of the associations between screen-based sedentary behaviors and mental health (19). The current study found that prolonged time (>2 h/day) on movie/TV viewing and social media using was associated with higher level of anxiety, however sedentary time on video gaming and recreational reading was not associated with anxiety. To some extent, our study supports recent findings regarding the differential effects of varied types and contents of screen time on mental health in children and adolescents (16, 17, 20). However, studies examining the association of different types of screen time with mental health in adulthood are still sparse. A study surveyed domain-specific sedentary behaviors in college students and found that leisure screen and non-screen based sedentary behavior was associated with higher trait anxiety and perceived stress (14). More studies are warranted to confirm the content-specific findings in the current study.

Interestingly, the current study showed that movie/TV viewing (>2 h) during weekdays, but not weekends, was associated with higher level of anxiety. The findings indicated a differential association of movie/TV viewing with anxiety between weekdays and weekends. Gibson and colleagues observed that weekday sitting time <8 h was associated with better perceived mental health (36). In contrast, Felez-Nobrega and colleagues observed that total self-reported sedentary time during weekend was associated with higher trait anxiety (14). These studies suggested a potential distinct association of sedentary behaviors during weekdays and weekend with mental health indicators in college students. However, it is obvious that more studies are needed to clarify the conflicting findings and understand the underlying mechanisms.

Based on the findings, the current study did not fully support the displacement hypothesis, which suggests that all screen-based sedentary time has negative effects on mental health (17). In this study, movie/TV viewing (>2 h during weekdays) and social media using (>2 h, during both weekdays and weekend) were positively associated with anxiety symptom, which indicated that the content of screen viewing may be crucial for developing anxiety symptom. These two types of screen-viewing all contain information which leads to upward comparison (i.e., to compare with others who perform better) (37), which may be discouraging and cause anxiety (38). Therefore, the findings at least partly support the social comparison theory and reinforcing theory. The current findings extended the literature by showing a content-specific association of screen-based sedentary behaviors with anxiety in college students.

To the best of our knowledge, the current study is the first to investigate the associations of device-assessed total sedentary time and different screen-based sedentary behaviors with mental health indicators in college students. However, some limitations of the study should also be acknowledged. Firstly, this study was cross-sectional in design. Therefore, no causal inference can be made based on the observed associations in the current study. It is therefore possible that higher level of anxiety leads to certain types of excessive screen-based sedentary behaviors. Secondly, the participants were recruited from one university and were about the same age, which may limit the generalization of the findings. Although college students are an important population to study, more studies with diverse samples are required to confirm the type-specific associations of screen time with mental health indicators.

## Conclusion

The study suggested that screen-based sedentary behaviors, but not device-measured total sedentary time, were associated with anxiety symptom among college students. The associations of screen-based sedentary behaviors with anxiety symptom varied by the type of screen time.

## Data availability statement

The raw data supporting the conclusions of this article will be made available by the authors, without undue reservation.

## Ethics statement

The studies involving human participants were reviewed and approved by Institutional Review Board for Human Research Protections. The patients/participants provided their written informed consent to participate in this study.

## Author contributions

TH: conceptualization, supervision, and project administration. TH, KZ, SL, and YY: methodology. SL, KZ, LK, and TH: validation. KZ and TH: formal analysis and writing and original draft preparation. SL, KZ, YZ, LK, and YY: resources. KZ and SL: data curation. TH, KZ, SL, YZ, LK, and YY: writing—review and editing. All authors have read and agreed to the published version of the manuscript.

## Conflict of interest

The authors declare that the research was conducted in the absence of any commercial or financial relationships that could be construed as a potential conflict of interest.

## Publisher's note

All claims expressed in this article are solely those of the authors and do not necessarily represent those of their affiliated organizations, or those of the publisher, the editors and the reviewers. Any product that may be evaluated in this article, or claim that may be made by its manufacturer, is not guaranteed or endorsed by the publisher.
